# Epidemic Hepatitis

**Published:** 1847-03

**Authors:** 


*Epidemic Hepatitis.*—To the Editor of the Boston Medical and Surgical Journal.—Sir,—Some atmospheric peculiarity has prevailed in the summer and autumn of the last year, which has had an uncommon influence upon the biliary organs. Since I have been engaged in the profession, we have had seasons when heat has been combined with wet, and seasons when heat has accompanied drought; but I have never seen any combination of atmosphere productive of such results have taken place in this vicinity the season just gone by. Sporadical instances of icterus have made their appearence more or less every year; but the jaundice as an epidemic, until the summer and autumn of last year, has never to my remembrance met my observation. In some instances it has been complicated with typhus fever, but in more, the organs merely concerned in the digestive process have suffered the effects of disease, and gave evidence of local derangement. The symptoms in different individuals were nearly similar. At the commencement of the disease a chilliness takes place, with a soreness about the gastric region on pressure, accompanied with flatulence and frebrile symptoms, and in some cases shooting pains extend through the side to the shoulder; the tongue gradually accumulating a light yellowish coat; the pulse not very highly excited, and in most cases the patient inclines to watchfulness and complains for want of sleep. The urinary discharges were scanty, and resembled bloody water from first to last. These were the more prominent symptoms showing themselves among the invalids the first few days, and then the skin began to show a light yellow tinge, which progressed to an orange color in a few days more, when it began to assume a more natural color, and at the end of two or three weeks the patient would resume his customary occupation if his disease was not complicated with typhus. A mild course of treatment succeeded with my patients. As few of them had much nausea, I seldom gave emetics, being apprehensive that the stomach was too sensitive and in too irritable a state to be benefited by them. Cathartics of jalap and calomel were given to vigorous males at the commencement of the disorder, and less drastic medicines to females and those with slender constitutions; and epispastics, in severe cases, were applied to the hepatic region.

This disease has prevailed on both sides of the Merrimac as far up as Manchester, and probably farther, and whether it is endemical to the region of the Merrimac is a question I should like to have answered by some of your correspondents more extensively acquainted with the extension of the disease than myself. My residence is about eight miles from the river. Two of my sons and several of my neighbors have been doing business at Merrimac the season past, and I believe every individual of them has returned home, sooner or later, sick with this singular epidemic. It has had the appearance in several instances of being contagious, but as a contagious jaundice would be on anomaly, or new thing under the sun, I desist from sporting in the marvellous, not having my "organs of marvellousness" fully developed.

To what this disease may be attributed, there are various conjectures; whether to a state of the atmosphere, a deficiency of water in wells, or to the stagnant waters of the reservoirs connected with the lakes at the head branches of the Merrimac, let off to supply the factories below, is a question. I have had some suspicions that the latter might be productive of a peculiar miasma which would specifically induce a diseased state of the biliary organs, but conjecture is all I can warrant. I was told by a gentleman from "up river," that the water from these reservoirs was green and slimy, which indicates its stagnated condition.

S. BROWN.

